# Massive retroperitoneal hematoma caused by intercostal artery bleeding after blunt trauma: a case report

**DOI:** 10.1186/s13019-024-02739-2

**Published:** 2024-04-18

**Authors:** Ran Zhu, Xiangtian Liu, Xiaoli Li, Liping Ye

**Affiliations:** Department of Intensive Care Medicine, The Affiliated Yantai Yuhuangding Hospital of Qingdao University, Yantai, China

**Keywords:** Retroperitoneal hematoma, Intercostal artery, Transcatheter arterial embolization, Trauma

## Abstract

**Background:**

The occurrence of massive retroperitoneal hematoma caused by intercostal artery bleeding is exceedingly uncommon.

**Case presentation:**

A middle-aged male presented to the hospital after a fall. Computed tomography scan revealed a massive retroperitoneal hematoma without any evidence of organ or major vessel rupture. The angiogram revealed extravasation from a branch of the twelfth intercostal artery, and successful transcatheter arterial embolization was performed on this specific artery.

**Conclusions:**

The possibility of intercostal artery rupture should be considered in cases of retroperitoneal hematomas, and accurate diagnosis can be achieved through imaging studies. Transcatheter arterial embolization represents an effective treatment modality.

## Background

Retroperitoneal hematoma is frequently associated with pelvic fractures, lumbar vertebral fractures, retroperitoneal organ rupture, or major retroperitoneal blood vessel rupture. Intercostal artery bleeding may result in hemothorax and chest wall or abdominal wall hematomas [1]. Here we present a case of massive retroperitoneal hematoma caused by the twelfth intercostal artery that was diagnosed clearly on computed tomography (CT) imaging and successfully treated with embolization.

## Case presentation

The patient, a 49-year-old male, presented to our hospital following a fall with the left subcostal area as the point of impact. Subsequently, he experienced severe lower back pain. He had no prior medical history. Upon admission, his vital signs were stable. A 10*10 cm subcutaneous bruise was observed on the left side, extending from the posterior axillary line to the area of the tenth to twelfth rib. There was evident tenderness at the left chest’s tenth to twelfth rib. Percussion on the lower left chest elicited a dull sound, indicating an underlying pathology. Palpation revealed a palpable mass in the left abdominal and lumbar region.

**Fig. 1:**
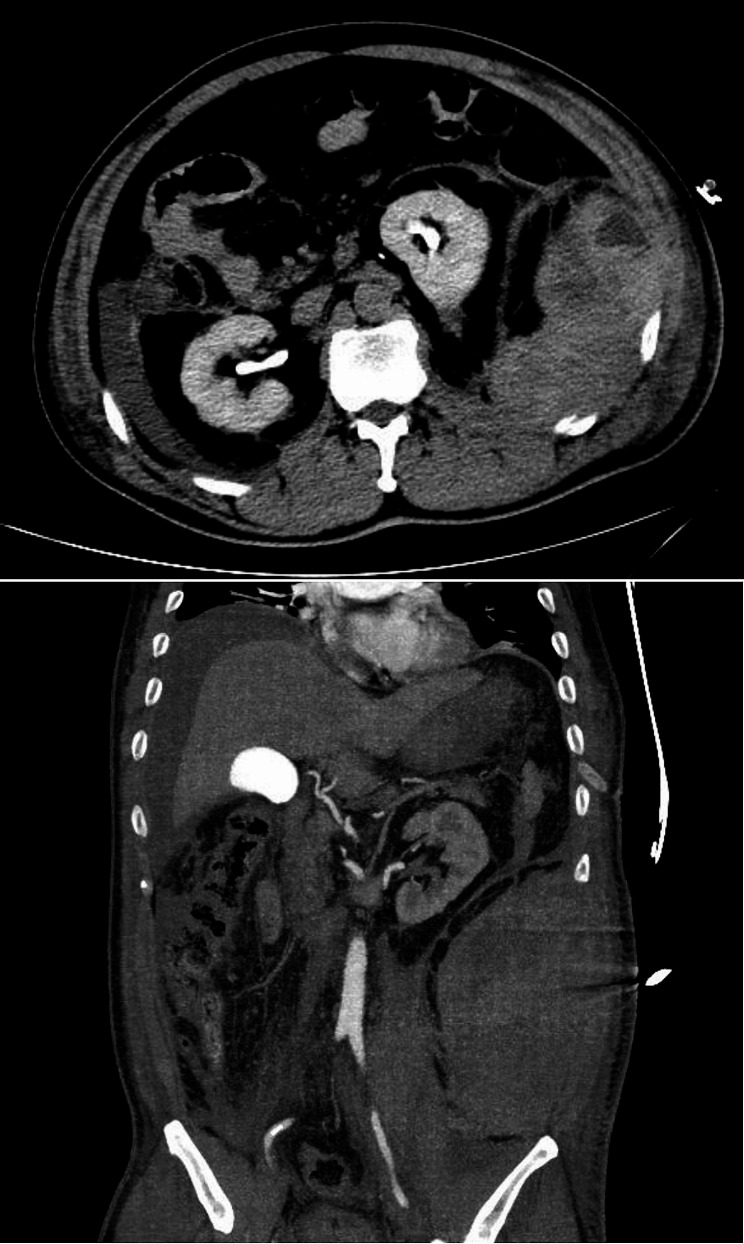
CT scan reveals the fractured end of the rib and retroperitoneal hematoma

Hemoglobin level measured at 61 g/L and hematocrit at 17.8%, while coagulation function remained within normal limits. Bedside abdominal ultrasound revealed a hypoechoic mass measuring approximately 20.2*10.5 cm beneath the skin in the left abdomen suggestive of hematoma formation. Enhanced CT scan of thoracoabdominal region demonstrated bilateral pleural effusion, fracture of the ribs X-XII on the left side, massive retroperitoneal hematoma measuring about 27.0*16.9*12.8 cm as well as pelvic hemorrhage (Fig. 1).

**Fig. 2:**
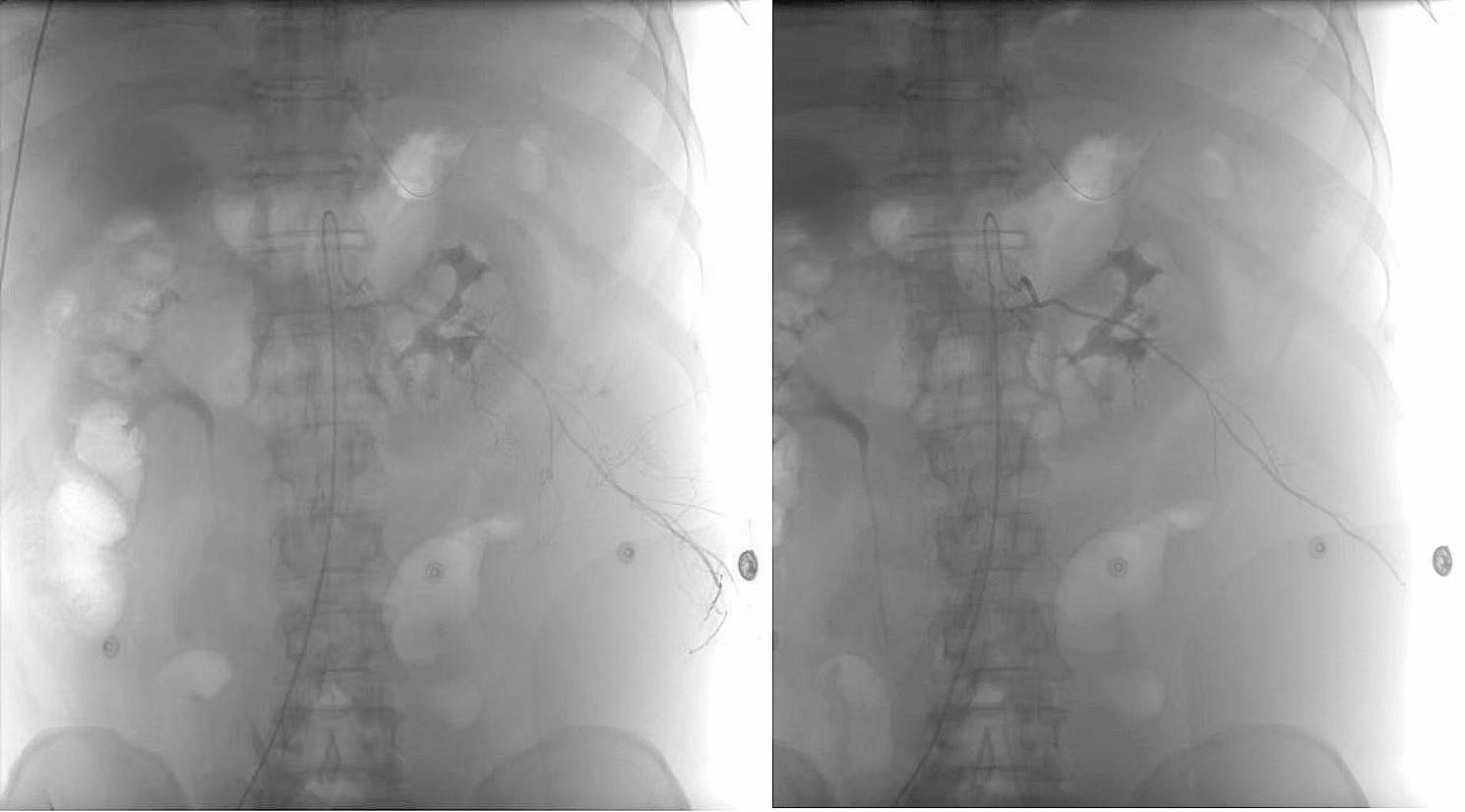
Left twelfth intercostal artery angiography showed extravasation, no extravasation was observed after embolization of the vessel

We promptly consulted with surgeons and interventional radiologists to discuss the treatment plan. Given the absence of significant organ bleeding and stable vital signs, we administered symptomatic treatment including red blood cell transfusion and thoracic catheter drainage. After 24 h, the patient’s abdominal pain intensified, accompanied by a drop in systolic blood pressure to 80mmHg, as well as decreased hemoglobin (48 g/L) and hematocrit levels (14.4%). Consequently, we opted for vascular angiography which revealed no extravasation from the abdominal aorta or its branches. However, selective arteriography of the intercostal artery revealed extravasation from a branch of the twelfth intercostal artery, implicating it as the source vessel for bleeding. Embolization was performed on this specific artery by injecting a mixture of Gelfoam pledgets and polyvinyl alcohol through a microcatheter (Fig. 2). After treatment, there was stabilization in hemoglobin levels, hematocrit levels, and blood pressure. Dynamic ultrasound evaluation demonstrated no enlargement of the retroperitoneal hematoma; hence percutaneous drainage under ultrasound guidance was planned once liquefaction occurred. The patient was discharged after two weeks of treatment and successfully resumed normal work activities during follow-up visits two months later.

## Discussion and conclusions

Peritoneal hematoma refers to hemorrhage occurring in the retroperitoneal space, which is a frequently encountered complication of abdominal and lumbar injuries. The primary etiologies include pelvic and lumbar vertebral fractures, followed by rupture of retroperitoneal organs such as the duodenum, pancreas, kidney, and bladder. Moreover, it can also be attributed to ruptured major blood vessels within the retroperitoneum resulting in bleeding.

Intercostal artery injury is predominantly caused by trauma [1], with blunt trauma resulting in rib fractures often accompanied by hemothorax, pneumothorax, pulmonary contusion, or less frequently, hematoma in the chest and abdominal wall [2]. To our knowledge, there have been no reports documenting massive retroperitoneal hematoma caused by intercostal artery bleeding following blunt abdominal trauma. In this particular case study, a patient presented with a substantial retroperitoneal hematoma subsequent to blunt abdominal trauma. Abdominal ultrasound and CT scans did not reveal any injuries to the abdominal organs or major blood vessels. However, angiography showed extravasation from a branch of the twelfth intercostal artery, implicating it as the source of hemorrhage. Following embolization of this specific artery, bleeding ceased and gradual improvement was observed in the patient’s condition.

In the majority of cases, symptoms resembling those of other common abdominal conditions are often observed in retroperitoneal hematoma, leading to frequent instances of underdiagnosis and neglect. This can subsequently result in severe complications and even mortality. Abdominal pain is the most prevalent presenting symptom, followed by a reduction in hemoglobin levels. Therefore, any unexplained decline in hemoglobin should raise suspicion regarding the possibility of a retroperitoneal hematoma [3]. Furthermore, certain subtle indications such as Grey Turner’s sign and Cullen’s sign may also serve as diagnostic clues for a retroperitoneal hematoma [4].

The diagnosis of retroperitoneal hematoma primarily relies on enhanced CT scanning, which serves to identify the presence and precise location of the hematoma while also providing guidance for treatment [5]. Notably, the detection of high-density shadows within the hematoma indicates extravasation of contrast agent, thereby suggesting active bleeding.

Studies have demonstrated that among patients with retroperitoneal hematoma, 50% may experience hemodynamic instability, only 17.9% necessitate vasopressor support. In the majority of cases, medical management serves as the primary treatment modality, with a minority requiring surgical intervention or endovascular embolization [3]. Transcatheter arterial embolization is considered a reliable and feasible therapeutic approach for controlling intercostal artery bleeding instead of thoracotomy [6]. Nevertheless, this technique’s efficacy varies due to its operator-dependency and carries a potential risk of postoperative pseudoaneurysm formation [7].

In conclusion, the occurrence of massive retroperitoneal hematoma caused by intercostal artery is an exceedingly rare and life-threatening condition. For patients presenting with hemodynamic instability, transcatheter arterial embolization is recommended as the primary treatment modality.

## Data Availability

No datasets were generated or analysed during the current study.

## Abbreviations

CT: Computed tomography
